# Iterative cycle of widely targeted metabolic profiling for the improvement of 1-butanol titer and productivity in *Synechococcus elongatus*

**DOI:** 10.1186/s13068-018-1187-8

**Published:** 2018-07-09

**Authors:** Artnice Mega Fathima, Derrick Chuang, Walter Alvarez Laviña, James Liao, Sastia Prama Putri, Eiichiro Fukusaki

**Affiliations:** 1 Department of Biotechnology, Graduate School of Engineering, Osaka University, 2-1 Yamadaoka, Suita, Osaka 565-0871 Japan; 2 Department of Chemical and Biomolecular Engineering, University of California, Los Angeles, 5531 Boelter Hall, 420 Westwood Plaza, Los Angeles, CA 90095 USA; 3 Microbiology Division, Institute of Biological Sciences, University of the Philippines Los, Banos, 4031 Philippines

**Keywords:** Cyanobacteria, Butanol, Widely targeted metabolomics, Rate-limiting step, Strain improvement

## Abstract

**Background:**

Metabolomics is the comprehensive study of metabolites that can demonstrate the downstream effects of gene and protein regulation, arguably representing the closest correlation with phenotypic features. Hence, metabolomics-driven approach offers an effective way to facilitate strain improvement. Previously, targeted metabolomics on the 1-butanol-producing cyanobacterial strain *Synechococcus elongatus* BUOHSE has revealed the reduction step from butanoyl-CoA to butanal, catalyzed by CoA-acylating propionaldehyde dehydrogenase (PduP), as a rate-limiting step in the CoA-dependent pathway. Moreover, an increase in acetyl-CoA synthesis rate was also observed in this strain, by which the increased rate of release of CoA from butanoyl-CoA was used to enhance formation of acetyl-CoA to feed into the pathway.

**Results:**

In the present study, a new strain (DC7) with an improved activity of PduP enzyme, was constructed using BUOHSE as the background strain. DC7 showed a 33% increase in 1-butanol production compared to BUOHSE. For a deeper understanding of the metabolic state of DC7, widely targeted metabolomics approach using ion-pair reversed-phase LC/MS was performed. Results showed a decreased level of butanoyl-CoA and an increased level of acetyl-CoA in DC7 compared to BUOHSE. This served as an indication that the previous bottleneck has been solved and free CoA regeneration increased upon the improvement of the PduP enzyme. In order to utilize the enhanced levels of acetyl-CoA in DC7 for 1-butanol production, overexpression of acetyl-CoA carboxylase (ACCase) in DC7 was performed by inserting the gene encoding an ACCase subunit from *Yarrowia lipolytica* into the *aldA* site. The resulting strain, named DC11, was able to reach a production titer of 418.7 mg/L in 6 days, compared to DC7 that approached a similar titer in 12 days. A maximum productivity of 117 mg/L/day was achieved between days 4 and 5 in DC11.

**Conclusions:**

In this study, the iterative cycle of genetic modification based on insights from metabolomics successfully resulted in the highest reported 1-butanol productivity for engineered *Synechococcus elongatus* PCC 7942.

**Electronic supplementary material:**

The online version of this article (10.1186/s13068-018-1187-8) contains supplementary material, which is available to authorized users.

## Background

1-Butanol is considered an important commodity chemical and advanced biofuel [1]; thus, the introduction of a CoA-dependent pathway to produce 1-butanol in various host organisms such as *Escherichia coli* [2, 3]*, Saccharomyces cerevisiae* [4]*, Pseudomonas putida*, *Lactobacillus brevis*, *Bacillus subtilis* [5, 6] and Cyanobacterium *Synechococcus elongatus* [7] has become a common strategy for microbial production. In addition, direct conversion of CO_2_ to produce valuable products using photosynthetic organisms has garnered significant interest for its prospective role in attaining a carbon neutral society [8–13]. Cyanobacteria, photosynthetic microorganisms, generally exhibit fast growth and genetic tractability, thus making them more advantageous as cell factories over eukaryotic algae and plants [14]. In relation to this, a cyanobacterial strain capable of producing 1-butanol was engineered by introducing a modified *Clostridial* CoA-dependent pathway [15, 16]. Initially, *Synechococcus elongatus* strain EL22 was engineered using an oxygen-sensitive enzyme, CoA-acylating butanal dehydrogenase (Bldh), for conversion of butanoyl-CoA to butanal and was able to produce a low titer of 29.9 mg/L of 1-butanol [15]. Since oxygen sensitivity of Bldh hindered 1-butanol production, it was replaced with an oxygen-tolerant enzyme (PduP) in the BUOHSE strain, which resulted in a significant increase in 1-butanol production [16].

In recent years, metabolomics-based approaches for strain improvement are widely being employed. This rapidly growing field, focusing on the whole metabolite profile of a biological system, provides valuable information that can be applied in numerous ways [3, 17–20]. By means of rapid detection of relevant metabolic perturbations, metabolomics can identify specific targets for strain improvement that may include identification of rate limiting steps in a production pathway, metabolite or product toxicity, cofactor imbalances, or depletion of metabolites consumed by alternative pathways [3]. Therefore, a deeper understanding of the metabolic state of 1-butanol-producing strains can facilitate strain improvement by further strain modifications.

Previously, a targeted metabolomics analysis focusing on the CoA-dependent pathway in 1-butanol producing *S. elongatus* strain was performed by our previous research group [18]. In this published work, strains that differ in enzymes that convert butanoyl-CoA to butanal were compared. The introduction of an oxygen tolerant (PduP) enzyme to replace the oxygen sensitive (BldH) enzyme was intended to increase the activity of this reaction in strain BUOHSE from base strain EL22. As a result, a major increase in 1-butanol titer was observed in BUOHSE compared to EL22. Consequently, we expected to observe a decrease in the amount of butanoyl-CoA in BUOHSE relative to EL22 due to a more effective conversion of butanoyl-CoA to butanal by the PduP enzyme. Surprisingly, the metabolome data indicated that the amount of butanoyl-CoA was comparable to that of EL22. Further analysis using kinetic profiling showed that the release of CoA resulting from the introduction of PduP in BUOHSE enabled free CoA regeneration that in turn led to the increased rate of acetyl-CoA synthesis. Here, the release of free CoA facilitated the reaction from pyruvate to acetyl-CoA, which was then used in the butanol pathway [18]. These facts strongly indicate that the reductive reaction of butanoyl-CoA to butanal should be modified further to improve 1-butanol productivity in the engineered cyanobacterial strain. In this work, we worked on further optimizing the PduP enzyme to facilitate a more effective conversion from butanoyl-CoA to butanal. In addition, since the previous analysis was only limited to the CoA-dependent pathway, there is a possibility that other rate-limiting steps are present within the 1-butanol-producing strain. Therefore, in the present study, widely targeted metabolic profiling was used to broaden the coverage of the metabolites analyzed, allowing for the identification of new rate-limiting steps in the 1-butanol biosynthesis pathway to ultimately improve both titer and productivity.

## Results and discussion

### Comparative metabolite analysis of EL22 and BUOHSE identified rate-limiting steps in 1-butanol biosynthesis

In this work, relative quantification approach for metabolomics was chosen over absolute quantification. It uses an internal standard to normalize the metabolite signal intensity. Since this procedure is experimentally less complicated and laborious, it allows for simultaneous profiling of a larger number of metabolites as compared to absolute quantification, thereby broadening the metabolite coverage [21, 22]. By using ion-pair reserved-phase liquid chromatography triple quadrupole mass spectrometer (IP-RP-LC/QqQ-MS) system, 74 metabolites (Additional file 1: Table S3) belonging to the central metabolism of *Synechococcus elongatus* strains EL22 and BUOHSE were successfully annotated.

**Fig. 1:**
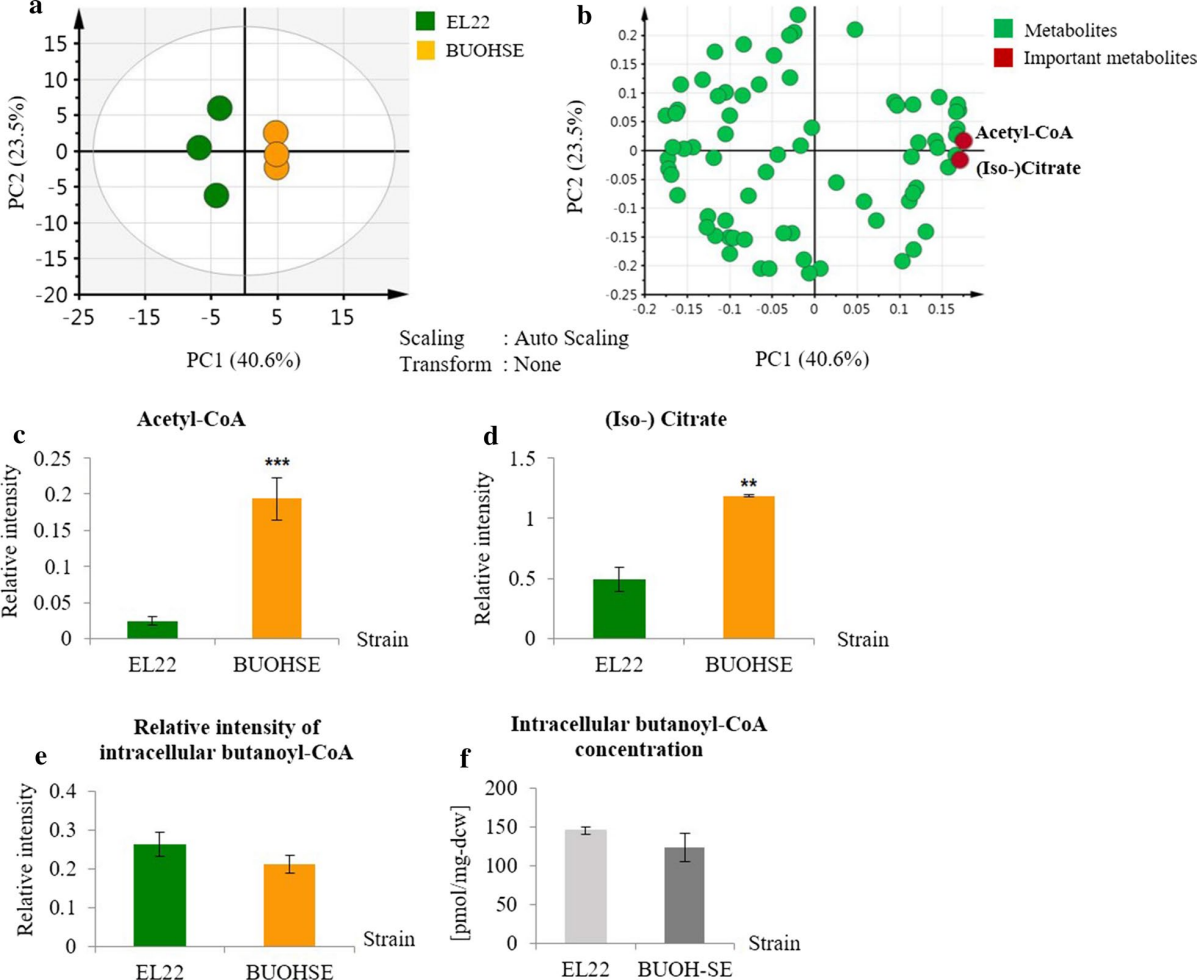
**a** Relative intensity of intracellular butanoyl-CoA measurements from widely targeted metabolic profiling of EL22 and BUOHSE. The y-axis represents the relative intensity of the metabolites, which was normalized to an internal standard. **b** Absolute intracellular concentration of butanoyl-CoA [pmol/mg-dry cell weight (dcw)kin EL22 and BUOHSE [18]. Samples were taken 67 h after IPTG induction. The error bars indicate standard deviations obtained from three replicates. **c** PCA score plot showing clear distinction between EL22 (in green circles) and BUOHSE (in yellow circles). **d** Loading scatter plot indicating metabolites that have influence on the clustering of the score plot. Relative intensity of two important metabolites: **e** acetyl-CoA and **f** (iso-) citrate. (Iso-) citrate in this study refers to iso-citrate and citrate as it is not possible to separate the two metabolites in our system. The y-axis represents the unit for the relative intensity of metabolites, which was normalized to an internal standard. Asterisks indicate significant differences in the strains (**p ≤ 0.01; ***p ≤ 0.001). All error bars indicate standard deviations obtained from three replicates

In order to confirm that the previous rate-limiting step can also be detected by using widely targeted analysis, the data were initially compared to the absolute quantification data from previously published results [18]. As mentioned previously, quantitative target analysis of acyl-CoAs suggested the accumulation of butanoyl-CoA in BUOHSE as the rate-limiting step in 1-butanol biosynthesis. Thus, the relative concentration of butanoyl-CoA in BUOHSE in comparison with EL22 was investigated. As shown in Fig. 1a, our results also showed comparable levels of butanoyl-CoA in EL22 and BUOHSE, illustrating a good agreement with the previous study [18]. Subsequently, we then employed Principle Component Analysis (PCA) to analyze which of the 74 annotated metabolites exhibited the most significant difference between the two strains.

Principal component analysis (PCA) is a well-known statistical technique used to determine variation and highlight important patterns in a data set [23]. The correlations between observations and its variables are easily seen by using this multivariate analysis. From the PCA score plots (Fig. 1a), a distinct separation of EL22 and BUOHSE along the first principal component (PC1) was observed. PC1 represented 40.6% of the total variance of the samples, while PC2 was 23.5%. Furthermore, PCA loading plot was examined in order to evaluate the factors contributing to the clustering seen on the score plots. The PCA loading plot showed that TCA cycle-related compounds such as acetyl-CoA and (iso-) citrate gave the most contribution for discriminating between the two strains (Fig. 1d). Relative intensity of TCA cycle intermediates was then investigated in order to get a better illustration. Results indicated that acetyl-CoA (Fig. 1e) and (iso-) citrate (Fig. 1f) accumulated in BUOHSE compared to EL22.

Significantly higher level of acetyl-CoA in BUOHSE compared to EL22 possibly occurred due to an increasing rate of acetyl-CoA synthesis, as explained in the previous work [18]. To validate this phenomenon, a widely targeted metabolome analysis of BUOHSE strain without *pduP* in comparison with BUOHSE strain was carried out. Results showed that the relative intensity of acetyl-CoA and (iso-) citrate in BUOHSE strain without *pduP* were significantly lower compared to BUOHSE, while butanoyl-CoA was significantly higher (Additional file 2: Figure S1). This suggested that without PduP, the conversion of butanoyl-CoA to butanal was hampered thus leading to the decrease in acetyl-CoA synthesis and (iso-) citrate formation. Hence, PduP is predicted to not only play a vital role in the conversion of butanoyl-CoA to butanal but also in free CoA regeneration, which is required for the function of the 1-butanol pathway. Therefore, this study also suggested that the improvement of PduP enzyme may be useful for enhancing 1-butanol production.

In addition, the higher level of (iso-) citrate observed in BUOHSE compared to EL22 indicated the possibility that the accumulated acetyl-CoA was used for the TCA cycle instead of the CoA-dependent 1-butanol pathway. Thus, diverting the increased acetyl-CoA pool towards the CoA-dependent 1-butanol pathway by improving the reaction from acetyl-CoA to malonyl-CoA seems to be a promising strategy for strain improvement.

### Enhancing 1-butanol titers by improving PduP enzyme activity

**Fig. 2:**
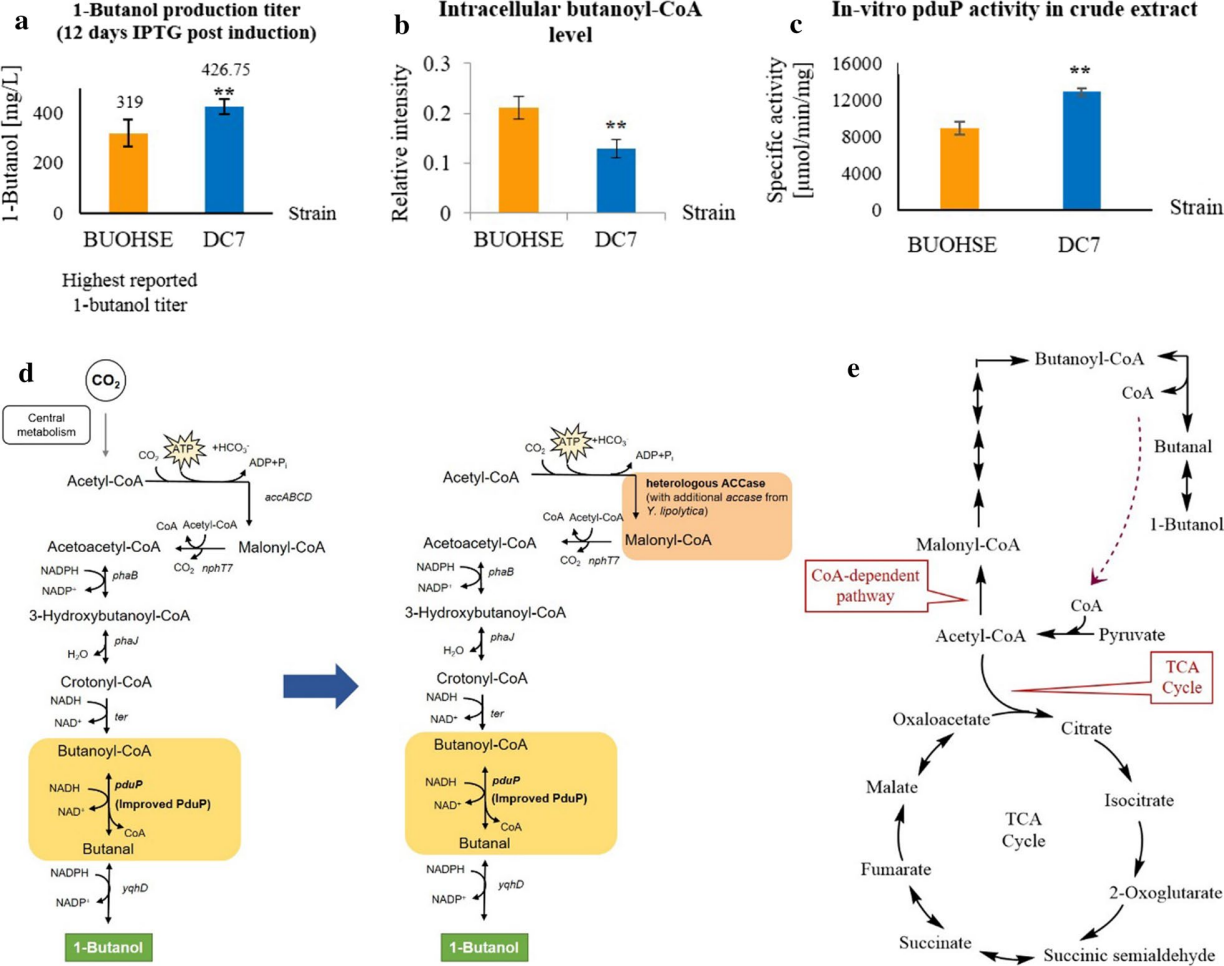
**a** In-flask 1-butanol titers from BUOHSE and DC7. Samples were taken 12 days after IPTG induction under light and aerobic conditions. **b** Relative intensity of intracellular butanoyl-CoA measurements in BUOHSE and DC7. **c** In-vitro specific activity of PduP in crude extract of BUOHSE and DC7. Asterisks indicate significant differences in the strains (**p ≤ 0.01). The error bars indicate standard deviations obtained from three replicates. **d** Schematic of genetic modification in synthetic 1-butanol biosynthesis pathway of DC7 and DC11. **e** Schematic representation of 1-butanol biosynthesis pathway and TCA cycle

To improve the reaction from butanoyl-CoA to butanal, PduP activity was improved in the BUOHSE background strain to generate strain DC7. Salis RBS calculator [24] is a useful tool for allowing modulation of RBS strength in various model organisms in order to control protein expression. In this study, it was used to design different RBS sequences to replace the original RBS upstream of *pduP* on the plasmid pSR3. Among several newly-constructed strains, 1-butanol production in DC7 outperformed the published strain BUOHSE [16] by 33% with a final titer of 426.75 mg/L in 12 days after IPTG induction (Fig. 2a). Specific enzyme activity in the crude extract was measured to validate if the PduP activity was in fact increased in DC7. A 1.4 fold increase in the PduP enzyme activity was observed in DC7 compared to BUOHSE (Fig. 2c). Furthermore, a significant decrease in intracellular butanoyl-CoA was also detected (Fig. 2b). Taken together, these results indicate that optimization of PduP activity effectively led to an improved conversion of butanoyl-CoA into butanal in DC7.

### Characterization of DC7 strain by widely targeted metabolic profiling

To gain a deeper understanding of the overall effect of the increased PduP activity in DC7 and to identify other targets for strain engineering, we applied the same widely targeted metabolic profiling strategy to compare BUOHSE and DC7.

**Fig. 3:**
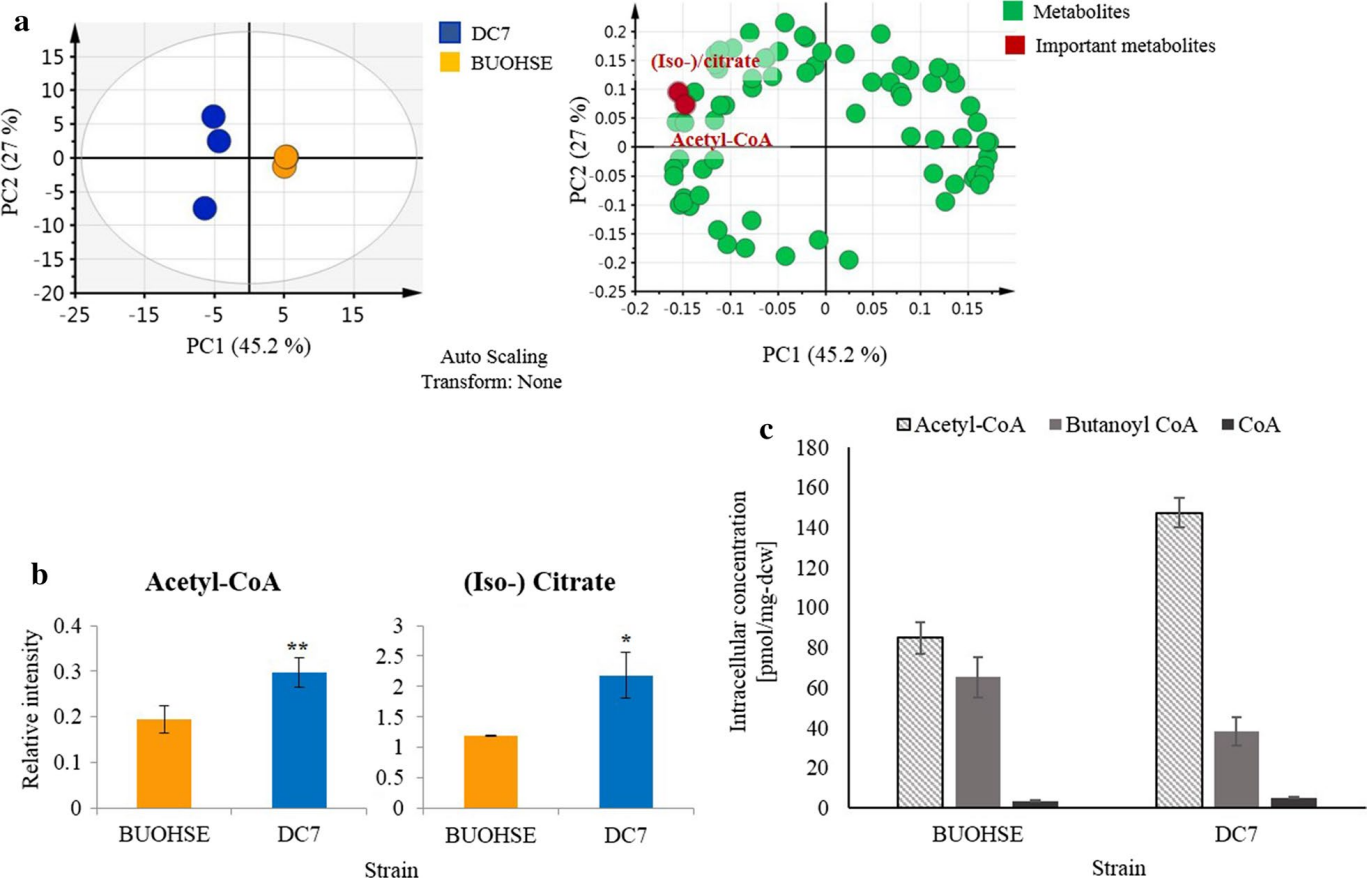
**a** PCA score plot and loading plot derived from metabolic profiling of BUOHSE and DC7. The score plot showed distinct separation of DC7 and BUOHSE, represented by blue and yellow circles, respectively. The loading plot revealed good correlation of acetyl-CoA and (iso-) citrate with the DC7 data set. **b** Relative intensity of intracellular acetyl-CoA and (iso-) citrate levels in BUOHSE and DC7. Asterisks indicate significant differences in the strains (*p ≤ 0.05; **p ≤ 0.01). **c** Intracellular concentration of acetyl-CoA, butanoyl-CoA and free CoA in BUOHSE and DC7. The error bars indicate standard deviation obtained from three replicates

Based on PCA analysis, acetyl-CoA and (iso-) citrate were found to be important metabolites for discriminating the two strains (Fig. 3a). Specifically, DC7 showed increased levels of acetyl-CoA and (iso-) citrate compared to BUOHSE (Fig. 3b). Interestingly, this result showed a similar tendency when EL22 and BUOHSE were compared, which also showed increased acetyl-CoA and (iso-) citrate. This also suggested that an increase in free CoA regeneration upon improvement of the PduP enzyme led to a higher accumulation of acetyl-CoA in DC7. Moreover, the enhanced level of (iso-) citrate in DC7 in comparison to BUOHSE strengthen the hypothesis generated from comparing EL22 and BUOHSE. It indicated that carbon from acetyl-CoA may be entering into the TCA cycle, which in turn may act as a drain of this 1-butanol precursor (Fig. 2e).

Since relative quantification indicates concentration relative to the internal standard, this method cannot be used for comparison of different metabolites within one strain. Hence, it could not be concluded whether the reaction to convert the accumulated acetyl-CoA to 1-butanol is a logical target for improvement as the concentration of acetyl-CoA cannot be compared to other CoA metabolites. Therefore, to know the absolute concentration of acetyl-CoA in comparison to other acyl-CoA metabolites, absolute quantification was carried out. Quantification of the absolute concentration of CoA-dependent pathway-related metabolites in the DC7 was performed by using ^13^C-labelled cyanobacterial cell extract as internal standard. Result showed that acetyl-CoA concentration was highly accumulated in the DC7 strain compared to other CoA-related metabolites (Fig. 3c). Thus, by diverting more acetyl-CoA into CoA-dependent pathway, further improvement of 1-butanol production might be achieved.

### Optimization of ACCase enzyme to divert enhanced level of acetyl-CoA towards 1-butanol formation

Carboxylation reaction of acetyl-CoA to malonyl-CoA is the direct downstream reaction of acetyl-CoA in the 1-butanol pathway and is catalyzed by the native ACCase complex [25]. This reaction serves as an essential component to many biosynthetic pathways [26], and is a notorious bottleneck in the production of a diverse set of compounds [27]. Therefore, as the next strategy for strain improvement, we focused on this reaction in order to enhance acetyl-CoA utilization. Modifications on the ACCase enzyme were carried out using DC7 as background strain. The ACCase enzyme in cyanobacteria, similar to majority of higher plants, is composed of multiple identical subunits [25, 28]. This complex structure of ACCase makes this enzyme difficult to modify or overexpress.

**Fig. 4:**
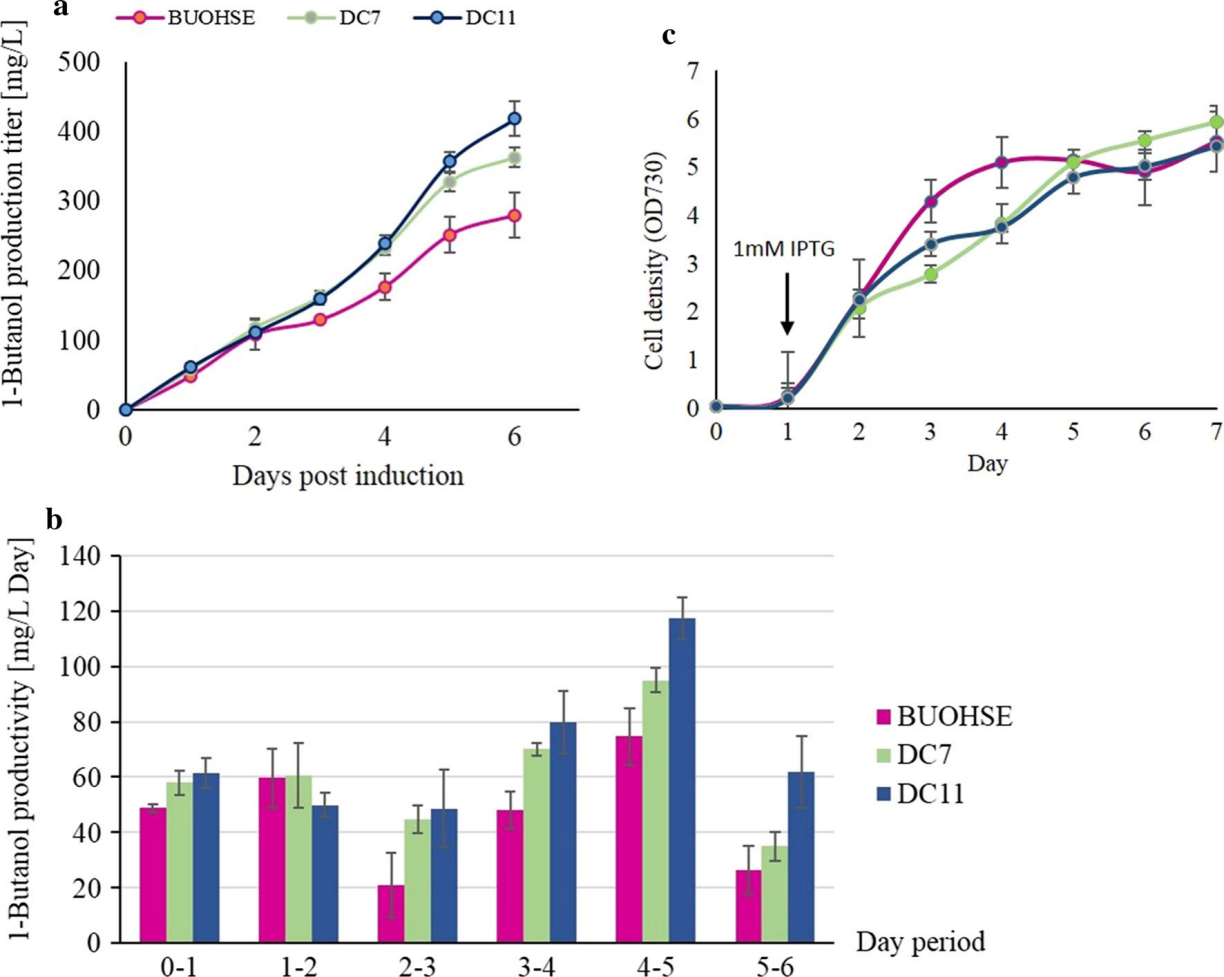
**a** 1-Butanol production and **b** cell density of *S. elongatus* BUOHSE, DC7, and DC11. Left bottom (**c**), daily productivity of 1-butanol by *S. elongatus* BUOHSE, DC7, and DC11. The error bars indicate standard deviations obtained from three replicates

However, several reports in *Yarrowia lipolytica* (*Y. lipolytica)* demonstrated the successful overexpression of a single subunit of ACCase for improvement of fuel-like molecules and oleochemicals production [29–32]. Moreover, several studies on the overexpression of ACCase in various microorganisms, such as *Escherichia coli* [33] and *Saccharomyces cerevisiae* [34] for production of valuable compounds, have also been published. Nevertheless, no studies have been reported on *Synechococcus elongatus*. Therefore, we attempted to increase the activity of ACCase by inserting a single subunit of *accase* gene from *Y. lipolytica* into the *aldA* site. In *Synechococcus elongatus* PCC7942, *aldA* encodes for alcohol dehydrogenase [30] that converts acetyl-CoA to acetaldehyde hence, disrupting this gene might also help to eliminate any unwanted consumption of acetyl-CoA. Based on this strategy, result showed that the DC11 strain (Fig. 2d), which contains *acc*ase from *Yarrowia lipolytica,* was able to reach a production titer of 418.7 mg/L in 6 days, while DC7 strain can reach a similar titer in 12 days (Fig. 4). This modification successfully achieved a maximum productivity of 117 mg/L/day between days 4 and 5, 57% higher compared to the best 1-butanol producing strain BUOHSE (74.5 mg/L/day) that was previously reported.

**Fig. 5:**
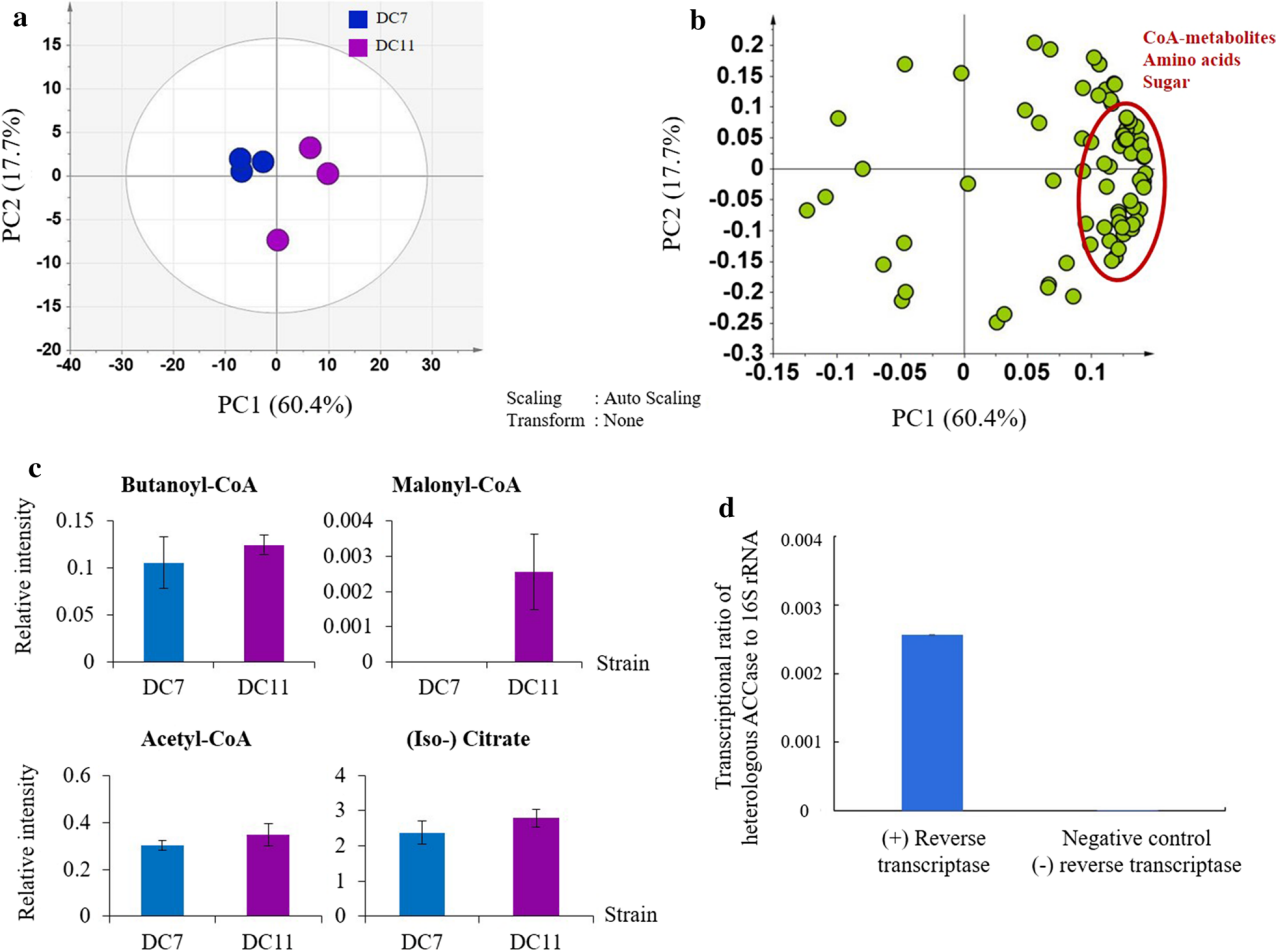
**a** PCA score plot and loading plot derived from DC7 and DC11. The score plot showed distinct separation of DC7 and DC11, represented by respective blue and purple circles. **b** The loading plot revealed CoA-dependent pathway-related metabolites, amino acids, and sugars had a positive contribution for separating DC11 with DC7. **c** Relative intensity of intracellular butanoyl-CoA, malonyl-CoA, acetyl-CoA and (iso-) citrate in DC7 and DC11. Asterisks indicate significant differences in the strains (*p ≤ 0.05; **p ≤ 0.01). **d** RT-PCR for heterologous *accase* expressed in *S. elongatus* PCC7942

To gain further insight into the metabolic perturbations that resulted from this modification, we employed widely targeted metabolomics to compare DC7 and DC11 strains. PCA results showed that CoA-related metabolites, amino acids, and sugars have a positive contribution in the separation of DC11 strain from DC7 strain (Fig. 5a, b). Since DC11 was developed from the DC7 strain, PduP enzyme profile for both strains was expected to be the same. Therefore, a comparable level of butanoyl-CoA in DC11 and DC7 was reasonably observed (Fig. 5c). Among the measured metabolites, malonyl-CoA showed a drastic increase in DC11 (Fig. 5c) thereby validating that the insertion of *accase* gene from *Y. lipolytica* was able to increase the ACCase activity. In addition, since there is no reliable assay platform to detect the activity of ACCase in crude extracts, we alternatively used reverse transcription polymerase chain reaction (RT-PCR) to confirm that the single unit of ACCase from *Y. lipolytica* was indeed transcribed in *S. elongatus* PCC7942 (Fig. 5d). However, in the metabolome data, we did not see any significant change in acetyl-CoA concentration or (iso-) citrate concentration. Acetyl-CoA pool is highly dynamic and can be affected by other factors, such as increasing rate of CoA recycling upon PduP improvement which resulted in an enhanced rate of acetyl-CoA synthesis that was previously mentioned in the previous study [18]. Moreover, simultaneous deletion of *aldA* may also aid in preventing acetyl-CoA degradation. Therefore, improvement of both ACCase and PduP in DC11 may conceivably improve the overall pathway leading to 1-butanol production. Furthermore, these results suggest that with further strain modifications to improve ACCase activity, additional improvements in butanol titers may be realized.

## Conclusions

Widely targeted metabolic profiling of EL22 and BUOHSE successfully confirmed that the reduction reaction from butanoyl-CoA to butanal, which is catalyzed by PduP enzyme, was the rate-limiting step in the CoA-dependent 1-butanol pathway. Increasing PduP activity was able to improve 1-butanol production in the DC7 strain by 33% compared to BUOHSE (the previously reported best 1-butanol producing strain). In addition, the accumulation of acetyl-CoA suggested that ACCase was also a rate-limiting step in the 1-butanol production pathway. Increasing the activity of ACCase enzyme through the insertion of *accase* from *Yarrowia lipolytica* in the DC7 strain resulted in an increase in 1-butanol productivity (DC11 strain). The DC11 strain reached a production titer of 418.7 mg/L in 6 days, while DC7 reached a similar titer in 12 days. The resulting strain developed in this study shows promise for future application in photosynthetic microbial based-1-butanol production and demonstrates the utility of metabolomics in metabolic engineering.

## Methods

### Cyanobacterial strains and plasmids

*Synechococcus elongatus* strains and plasmids used in this study are listed in Table 1. Cyanobacterial transformation and plasmid construction are described in our previous work [15, 16]. Salis calculator [35, 36] is used as a tool to generate different RBS sequences and replace the original RBS for *pduP* on the plasmid pSR3. Strain EL9 [7] was transformed with plasmid pDC304 containing a synthetic RBS sequence TCACAAAATACTTACCAACAAAGGAGGATCCC in front of *pduP*, resulting in a new strain DC7. Plasmid pDC331 encoded by *accase* gene from *Yarrowia lipolytica* was introduced in strain DC7 into the *aldA* site and the resulting strain DC11 was selected by 5 µg/mL gentamicin on the plates. Full segregation of each strain was verified through PCR (Additional file 2: Figure S2).

**Table 1:** Strains and plasmids used in this study

	Genotype characteristic	References
Cyanobacteria strain		
PCC 7942	Wild-type *Synechococcus elongatus* PCC 7942	–
EL22	P_Trc_:: His-tagged *T. denticola ter* integrated at NSI and PL_*lacO1*_:: *nphT7, bldh, yqhD, phaJ, phaB* integrated at NSII in PCC 7942 genome	[15]
BUOHSE	P_Trc_:: His-tagged *T. denticola ter* integrated at NSI and PL_lacO1_:: *nphT7, pduP*_*S. enterica*, *yqhD, phaJ, phaB* integrated at NSII	[16]
BUOHSE without *pduP*	P_Trc_:: His-tagged *T. denticola ter* integrated at NSI and PL_lacO1_:: *nphT7, phaJ, phaB* integrated at NSII	[18]
DC7	P_Trc_:: His-tagged *T. denticola ter* integrated at NSI and PL_lacO1_:: *nphT7, PduP*_*S. enterica*, *yqhD, phaJ, phaB* integrated at NSII, synthetic RBS TIR: 522,266.72 in front of *pduP*	This work
DC11	P_Trc_:: His-tagged *T. denticola ter* integrated at NSI and PL_lacO1_:: *nphT7, pduP*_*S. enterica*, *yqhD, phaJ, phaB* integrated at NSII, synthetic RBS TIR: 522,266.72 in front of *pduP* PL_lacO1_: *ACCase* integrated at *aldA*	This work
Plasmid		
pSR3	Kan^R^; NSII targeting; PL_lacO1_:: *nphT7, yqhD, pduP, phaJ, phaB*	[16]
pEL256	Kan^R^; NSII targeting; PL_lacO1_:: *nphT7, phaJ, phaB*	[18]
pDC304	Kan^R^; NSII targeting; PL_lacO1_:: *nphT7, yqhD*, synthetic RBS for *pduP, phaJ, phaB*	This work
pDC331	Gen^R^; aldA targeting; PL_lacO1_: *ACCase Yarrowia lipolytica* integrated at *aldA*	This work

*nphT7* (*Streptomyces* sp. Strain CL190), acetoacetyl-CoA synthase; *bldh* (*C. saccharoperbutylacetonicum*), CoA-acylating butanal dehydrogenase; *yqhD* (*E. coli*), NADPH-dependent alcohol dehydrogenase; *phaJ* (*A. caviae*), (*R*)-specific crotonase; *phaB* (*R. eutropha*), acetoacetyl-CoA reductase; *pduP* (*S. enterica*), CoA-acylating propionaldehyde dehydrogenase; *ACCase* (*Yarrowia lipolytica*), acetyl-CoA carboxylase. *Kan*^*R*^ kanamycin resistance, *Gen*^*R*^ gentamycin resistance

### Culture medium and growth conditions

All cyanobacterial strains were cultured at 30 °C, under constant illumination of 50 µmol/photon/m^2^/s in a temperature-controlled chamber. Liquid cultures were grown in modified BG-11 medium containing 50 mM NaHCO_3_ in 300 mL screw cap flasks with continuous shaking at 120 rpm as previously described [18]. In the case of mutants, 20 mg/L spectinomycin and 10 mg/L kanamycin were added to the medium. For pre-culture, a loopful of cells from solid medium were inoculated into a 20 mL liquid medium to an OD_730_ of 1.5–2.0. For the main culture, cells were inoculated in a 50 mL BG-11 liquid medium with an initial density of OD_730_ = 0.04. Feeding with 5 mL of fresh BG-11 medium containing 500 mM NaHCO_3_ was done every 2 days until sampling time. IPTG and antibiotics were also added during feeding time.

### Sampling and extraction

Sampling by fast filtration was done at 67 h after IPTG induction as previously described [18]. Briefly, cell culture equivalent to 5 mg dry cell weight was filtered using a 0.2 μm pore size Omnipore membrane filter disc (Millipore, USA). Subsequently, the filtered cells were washed with pre-cooled 70 mM NH_4_CO_3_. The membrane filter containing the cells was placed on pre-chilled aluminum block to arrest cellular metabolic activity. Samples were stored in 15 mL centrifuge tubes at − 80 °C until extraction.

Extraction of intracellular metabolites was done using the liquid–liquid extraction protocol with some modifications [17]. One milliliter of mixed solvent (CH_3_OH:CHCl_3_:H_2_O, 5:2:2, v/v) containing the internal standard [5 ppm of (+)-10-camphorsulfonic acid] was added to the sample tube. A freeze and thaw procedure consisting of incubation at − 80 °C for 1 h followed by − 30 °C for 30 min was done. The sample tube was then mixed by vortex for 30 s and sonicated for 10 s. This procedure was repeated two more times. Two milliliter of suspension was divided equally into 2 mL microfuge tubes and added with 200 μL of water. Centrifugation at 10,000*g* for 5 min at 4 °C was performed to separate the polar and nonpolar phases. Eight hundred microliter of the resulting polar phase was transferred into a new 1.5 mL microfuge tube through filtering with a 0.20 μm Millex-LG filter (Millipore, USA). To concentrate the samples, the filtered supernatant was subjected to centrifugal concentration for 2 h using a VC-96R Spin Dryer Standard (Taitec, Tokyo, Japan). Identical samples were then combined before overnight lyophilization in a VD-800F Freeze Dryer (Taitec, Tokyo, Japan). Lyophilized samples were stored at − 80 °C until LC/MS/MS analysis.

In the case of extracellular metabolites extraction, 1 mL of culture medium was collected and centrifuged at 16,000*g* for 5 min at 4 °C. The supernatant was transferred to a new 1.5 mL microfuge tube. Samples were stored at − 30 °C until GC/FID analysis.

### Absolute quantification of CoA-related metabolites

The intracellular concentration of CoA, acetyl-CoA, and butanoyl-CoA was determined using ^13^C-isotope labelling experiment following the protocol used in the previous study with minor modifications [18]. Fully ^13^C-labeled cell extracts of BUOHSE and DC7 strains were prepared. Cells were cultivated as described previously except that NaH^13^CO_3_ (> 98 atom % ^13^C, Cambridge Isotope Laboratories, Inc., USA) was used instead of NaHCO_3_. Sampling was performed 3 days after IPTG induction in the same way as described above except that pre-cooled deionized water was used as washing solvent instead of NH_4_CO_3_. Cells were extracted as described above except without the freeze-drying step. Extraction was repeatedly conducted for four times. After samples were concentrated for 2 h using centrifugal concentration, all samples were combined in a 15 mL centrifuge tube. This ^13^C-labelled cell extract was used as an internal standard. Six point calibration curve was constructed for each metabolite using peak area ratios of U-^12^C to U-^13^C, as described previously [18]. The detailed description of calibration curve for each metabolite is listed in Additional file 1: Table S1. Analysis mode was multiple reaction monitoring (MRM) mode in IP-RP-LC/QqQ-MS system (Additional file 1: Table S4).

### Ion-pair reversed-phase LC/QqQ-MS analysis

The freeze-dried samples were dissolved in 30 µL of ultrapure water for RP-IP-LC/QqQ-MS analysis using a Shimadzu Nexera UHPLC system coupled with LCMS 8030 plus (Shimadzu Co., Japan) with an L-column 2 ODS (150 mm × 2.1 mm, 3 μm, Chemicals Evaluation and Research Institute, Japan) as previously described [17]. Analysis was performed using 10 mM tributylamine-15 mM acetic acid in water as mobile phase A and methanol as mobile phase B. The gradient concentration of the mobile phase with flow rate of 0.2 mL/min were as follows: start from 0% B at 0–1 min, increased to 15% B at 1.0–1.5 min, then held until 3.0 min, then increased to 50 and 100% B from 3.0 to 8.0 min, and 8.0 to 10.0 min, respectively; held 100% B until 11 min, decreased to 0% B from 11 to 11.50 min, and at last held 0% B until 17 min. The column oven temperature was 45 °C and the analysis mode was in negative ion mode. The mass spectrometer conditions were set at the following conditions: the desolvation line temperature was 250 °C, the nebulizer gas flow was 2 L/min, the drying gas flow was 15 L/min, and the heat block temperature was 400 °C and analysis mode was multiple reaction monitoring (MRM) mode (Additional file 1: Table S2).

### 1-Butanol analysis

Alcohols were quantified by a GC-2010 system (Shimadzu) equipped with a flame ionization detector and an AOC-20i/s autoinjector (Shimadzu) using a GL Science (Tokyo, JAPAN) InertCap Pure-WAX capillary column (30 m, 0.25 mm i.d., 0.25 μm film thickness). Isobutanol was added into the sample as an internal standard. The injector was maintained at 225 °C. The column temperature was initially held at 40 °C for 1 min and raised with a gradient of 15 °C/min until 120 °C and held for 1 min, then raised with a gradient of 50 °C/min until 250 °C and held for 5 min. Nitrogen was used as the carrier gas with a column flow rate of 1.90 mL/min (linear velocity 40.0 cm/s).

### Data analysis

The raw data set from LC/MS/MS analysis was converted to an analysis base file (.abf) format using a converter developed by Reifycs Inc., Tokyo, JAPAN. MRMPROBS 2.19 [37] was used for peak picking and calculating the peak area. The detected peaks were also confirmed manually by using Lab solution (Shimadzu Co., Japan). Subsequently, the data matrix was subjected to multivariate analysis. To make the data easy to visualize, SIMCA-P+ version 13 (Umetrics, Umeå, Sweden) was used for constructing the PCA (principal component analysis) plots. All data were standardized to autoscale: mean was 0, variance was 1. In addition, a Student’s *t* test analysis was performed using MS Excel to determine whether two sets of data were significantly different from each other.

### PduP enzyme assay

Enzyme assays for PduP activity in the cyanobacterial crude extracts were performed as described in the previous work [16]. Cyanobacterial crude extracts were prepared by harvesting the fresh cultures after IPTG induction. The details of preparation of cell extract and the incubation procedure have been described elsewhere [7]. A Bio-Tek Power-Wave XS microplate spectrophotometer was used to monitor the decrease of absorbance at 340 nm, corresponding to the consumption of NADH. The reaction mixture contained 1 mM dithiothreitol (DTT), 500 μM NADH, 500 μM butanoyl-CoA, and 50 mM phosphate buffer (pH 7.15). The enzymatic reaction was started after the addition of the crude extract.

### RNA extraction and reverse transcription polymerase chain reaction (RT-PCR)

Strain DC11 grown in BG11 with 50 mM sodium bicarbonate under light condition (50 µE/m^2^s) was induced by 1 mM IPTG when OD_730_ reached between 0.4 and 0.6. The cultures were harvested 24 h after IPTG induction. The total RNA was extracted by using the RiboPure RNA Purification kit (Invitrogen), following the manufacturers’ protocol. The isolated RNA was further treated with DNase I (Invitrogen) for 30 min at 37 °C. The reagents for reverse transcription PCR was performed by using the iTaq Universal SYBR Green One-Step kit (BIO-RAD). The primers for the RT-PCR were designed by the NCBI/Primer-Blast tools. The forward primer 5′-GGAATCGCTAGTAATCGCA-3′ and reverse primer 5′-GCTACCTTGTTACGACTTCA-3′ were used to amplify 16S rRNA as a control. The forward primer 5′-TTGTCACCACTGAGATTGAG-3′ and the reverse primer 5′-TATCCTTGTAGGCTCGAGAA-3′ were used to amplify the heterologous *acetyl*-*CoA carboxylase* (*accase*) from *Yarrowia lipolytica*. RT-PCR was conducted by the following condition: 50 °C for 10 min, 95 °C for 1 min, 40 cycles of 95 °C for 10 s and 60 °C for 30 s. The melting curve was obtained from 65 °C to 95 °C by the increase of 0.5 °C. The cycle threshold (C_t_) value was acquired and analyzed by the CFX Manager software (BIO-RAD).

## Additional files

**Additional file 1: Table S1.** Calibration curves of acetyl-CoA, butanoyl-CoA, and free CoA, was acquired by using reversed phase- ion pairing- liquid chromatography- mass spectrometry (RP-IP-LC/QqQ-MS) system. The horizontal axis is the area ratio of monoisotopic peak to uniformly ^13^C-labeled peak and vertical axis is naturally labeled standard amount in pmol/tube. U-^13^C / (U-^13^C+U-^12^C) means the ratio of U-^13^C to (U-^13^C + U-^12^C) peak area in internal standard [18]. **Table S2**. Multiple reaction monitoring (MRM) transitions for widely targeted analysis in RP-IP-LC/QqQ-MS system. **Table S3**. Annotated metabolites in widely targeted analysis (74 metabolites in samples were annotated using method for 121 metabolites MRM transitions, described in **Table S2**). **Table S4**. Multiple reaction monitoring (MRM) transitions for absolute quantification of CoA-related metabolites by using RP-IP-LC/QqQ-MS system [18].

**Additional file 2: Figure S1.** Relative intensity of intracellular butanoyl-CoA, acetyl-CoA, and (iso-) citrate in BUOHSE and BUOHSE without *pduP*. Asterisks indicate significant differences in the strains (*: p ≤ 0.05; **: p ≤ 0.01). **Figure S2**. Colony PCR results for the segregation test in BUOHSE, DC7, and DC11 strain, W. T. (wild type strain), NSI (neutral site I), NSII (neutral site II). **Figure S3**. 1-Butanol production and cell density of *S. elongatus* obtained with the different RBS sequences.

## Abbreviations

*nphT7*: acetoacetyl-CoA synthase
*bldh*: CoA-acylating butanal dehydrogenase
*yqhD*: NADPH-dependent alcohol dehydrogenase
*phaJ*: (*R*)-specific crotonase
*phaB*: acetoacetyl-CoA reductase
*pduP*: CoA-acylating propionaldehyde dehydrogenase
*ACCase*: acetyl-CoA carboxylase
Kan^R^: kanamycin resistance
Gen^R^: gentamycin resistance
IP-RP-LC/QqQ-MS: ion-pair reversed-phase liquid chromatography triple quadrupole mass spectrometer
PCA: principal component analysis
RBS: ribosome binding site
IPTG: isopropyl β-d-1-thiogalactopyranoside
BG-11: blue-green 11
RT-PCR: reverse transcription polymerase chain reaction
