# Supplementary material for: Iterative cycle of widely targeted metabolic profiling for the improvement of 1-butanol titer and productivity in Synechococcus elongatus
Source: Biotechnol Biofuels. 2018 Jul 9;11:188. doi: 10.1186/s13068-018-1187-8 (PMC6036673; doi:10.1186/s13068-018-1187-8)
Supplement: Supplementary file 1 — Additional file 1: Table S1. Calibration curves of acetyl-CoA, butanoyl-CoA, and free CoA, was acquired by using reversed phase- ion pairing- liquid chromatography- mass spectrometry (RP-IP-LC/QqQ-MS) system. The horizontal axis is the area ratio of monoisotopic peak to uniformly 13C-labeled peak and vertical axis is naturally labeled standard amount in pmol/tube. U-13C / (U-13C+U-12C) means the ratio of U-13C to (U-13C + U-12C) peak area in internal standard [18]. Table S2. Multiple reaction monitoring (MRM) transitions for widely targeted analysis in RP-IP-LC/QqQ-MS system. Table S3. Annotated metabolites in widely targeted analysis (74 metabolites in samples were annotated using method for 121 metabolites MRM transitions, described in Table S2). Table S4. Multiple reaction monitoring (MRM) transitions for absolute quantification of CoA-related metabolites by using RP-IP-LC/QqQ-MS system [18]. [file 13068_2018_1187_MOESM1_ESM.docx]

# **Additional file 1**

Table S1

Calibration curves of acetyl-CoA, butanoyl-CoA, and free CoA, was acquired by using reversed phase- ion pairing- liquid chromatography- mass spectrometry (RP-IP-LC/QqQ-MS) system. The horizontal axis is the area ratio of monoisotopic peak to uniformly ^13^C-labeled peak and vertical axis is naturally labeled standard amount in pmol/tube. U-^13^C / (U-^13^C+U-^12^C) means the ratio of U-^13^C to (U-^13^C + U-^12^C) peak area in internal standard [18].

| Metabolite | Range [pmol/tube] | Equation | R^2^ | U-^13^C / (U-^13^C+U-^12^C) |
| --- | --- | --- | --- | --- |
| Acetyl-CoA | 1 ~ 243 | y = 7.2294x + 6.2919 | 0.992 | 1.00 |
| Butanoyl-CoA | 1 ~ 243 | y = 42.733x + 6.4882 | 0.992 | 0.98 |
| CoA | 0.25 ~ 8 | y = 0.225x + 0.9567 | 0.938 | 0.91 |

Table S2 Multiple reaction monitoring (MRM) transitions for widely targeted analysis in RP-IP-LC/QqQ-MS system

| Metabolite | Precursor ion m/z | Product ion m/z | Retention Time (minute) | Target Collision Energy (V) |
| --- | --- | --- | --- | --- |
| Arginine | 173.05 | 131.05 | 1.272 | 15 |
| Lysine | 145.1 | 97.05 | 1.275 | 13 |
| Histidine | 154.05 | 93 | 1.277 | 21 |
| 4-Aminobutyrate | 162.05 | 102 | 1.465 | 8 |
| Serine | 104.05 | 74.1 | 1.653 | 16 |
| Asparagine | 131.05 | 113.05 | 1.659 | 15 |
| Glutamine | 145.1 | 127.05 | 1.691 | 18 |
| Threonine | 118.05 | 74.05 | 1.702 | 16 |
| Hydroxyproline | 190.05 | 130.05 | 1.709 | 10 |
| Hexose | 179.05 | 89 | 1.743 | 19 |
| 2-Aminobutyrate | 162.05 | 102 | 1.817 | 8 |
| Cysteine | 239.05 | 120.1 | 1.825 | 13 |
| Trehalose | 341.05 | 89.1 | 1.885 | 23 |
| Proline | 174.05 | 114 | 1.898 | 10 |
| Sucrose | 341.05 | 89.1 | 2.074 | 23 |
| Valine | 176.05 | 116.05 | 2.201 | 10 |
| Cytidine | 302.05 | 242 | 2.337 | 10 |
| Pyridoxamine-5Phospate | 247.05 | 230 | 2.453 | 11 |
| Methionine | 148.05 | 47.05 | 2.767 | 14 |
| Guanine | 150.05 | 133.05 | 3.126 | 21 |
| Hypoxanthine | 135.05 | 92 | 3.546 | 28 |
| Tyrosine | 180.05 | 163.05 | 3.554 | 18 |
| Adenine | 134.1 | 107.05 | 3.589 | 22 |
| Isoleucine | 190.05 | 130.05 | 3.638 | 10 |
| Leucine | 190.05 | 130.05 | 3.963 | 10 |
| Xanthine | 151.05 | 108.05 | 3.978 | 18 |
| Glutamate | 146.05 | 102.05 | 4.284 | 15 |
| Uridine | 243.05 | 110.05 | 4.325 | 17 |
| Aspartate | 132.05 | 88.05 | 4.426 | 14 |
| Inosine | 267.05 | 135.05 | 4.566 | 23 |
| Thymine | 125.05 | 42 | 4.575 | 18 |
| Guanosine | 282.1 | 150.05 | 4.605 | 21 |
| Urate | 167.1 | 124.05 | 4.699 | 15 |
| Shikimate | 173.05 | 93 | 4.854 | 17 |
| Adenosine | 266.1 | 134.05 | 4.975 | 25 |
| Glycerate | 105.05 | 75.05 | 5.097 | 13 |
| Thymidine | 301.1 | 241 | 5.151 | 10 |
| Phenylalanine | 164.05 | 147.05 | 5.208 | 18 |
| Glycolate | 75.05 | 47.05 | 5.335 | 13 |
| Glyoxylate | 73 | 73 | 5.754 | 5 |
| G6P | 259.05 | 97 | 6.303 | 17 |
| Disaccharide-Phosphate | 421.1 | 79.05 | 6.372 | 40 |
| Mn6P | 259.05 | 97 | 6.535 | 17 |
| Sor6P | 261.05 | 97 | 6.593 | 23 |
| Pyroglutamate | 188.05 | 128 | 6.618 | 12 |
| Tryptophan | 203.1 | 116.05 | 6.675 | 18 |
| R5P | 229.05 | 97 | 6.691 | 13 |
| Succinic Semialdehyde | 101.05 | 57 | 6.697 | 13 |
| Lactate | 89.05 | 43 | 6.779 | 14 |
| S7P | 289.1 | 97 | 6.803 | 21 |
| F6P | 259.05 | 97 | 6.823 | 17 |
| Ara5P | 229.05 | 97 | 6.982 | 13 |
| G1P | 259.05 | 79.05 | 7.062 | 28 |
| α-Glycerophosphate | 171.05 | 79.05 | 7.063 | 18 |
| TPP | 424.1 | 302.05 | 7.103 | 16 |
| NAD | 662.1 | 540.1 | 7.188 | 18 |
| GAP | 169.05 | 97 | 7.288 | 12 |
| Orotate | 155.05 | 111.05 | 7.301 | 14 |
| Ru5P | 229.05 | 97 | 7.459 | 13 |
| CMP | 322.1 | 79.05 | 7.488 | 28 |
| β- Glycerophosphate | 171.05 | 79.05 | 7.597 | 18 |
| MEP | 215.05 | 79.05 | 7.623 | 27 |
| F1P | 259.05 | 97 | 7.651 | 17 |
| Pyruvate | 87.05 | 43 | 7.782 | 11 |
| R1P | 229.05 | 79.05 | 7.812 | 25 |
| UMP | 323.1 | 79.05 | 7.951 | 36 |
| AICAR | 337.1 | 79.05 | 8.001 | 37 |
| GMP | 362.1 | 79.05 | 8.031 | 26 |
| IMP | 347.05 | 79.05 | 8.049 | 40 |
| DHAP | 169.05 | 97 | 8.085 | 12 |
| TMP | 321.1 | 195.05 | 8.572 | 20 |
| AMP | 346.1 | 79.05 | 8.618 | 38 |
| Pantothenate | 218.05 | 88 | 8.814 | 17 |
| Nicotinate | 122.05 | 78 | 8.818 | 16 |
| cAMP | 328.1 | 134.05 | 9.143 | 27 |
| Succinate | 117.05 | 73 | 9.568 | 15 |
| Carbamoyl-P | 140.05 | 79.05 | 9.569 | 22 |
| Glutathione | 306.05 | 143.05 | 9.571 | 20 |
| Malate | 133.05 | 115 | 9.848 | 17 |
| UDP-Glu | 565.05 | 323.05 | 9.878 | 27 |
| XMP | 363.1 | 211.05 | 9.961 | 20 |
| CDP | 402.1 | 79.05 | 10.021 | 42 |
| Acetyl-P | 139 | 79.05 | 10.045 | 14 |
| 2OG | 145.1 | 101.05 | 10.046 | 10 |
| ADP-Glu | 588.05 | 346.05 | 10.069 | 23 |
| Fumarate | 115.05 | 71 | 10.128 | 10 |
| GDP | 442.1 | 79.05 | 10.131 | 45 |
| 6PGA | 275.05 | 177.05 | 10.154 | 16 |
| UDP | 403.1 | 159 | 10.176 | 28 |
| 3PGA | 185.05 | 97 | 10.181 | 16 |
| Shikimate-3P | 253.05 | 97 | 10.224 | 13 |
| NADH | 664.1 | 79.05 | 10.238 | 57 |
| NADP | 742.1 | 620.1 | 10.329 | 18 |
| ADP | 426.1 | 79.05 | 10.353 | 46 |
| SBP | 369.1 | 97 | 10.374 | 27 |
| Citrate | 191.05 | 87 | 10.399 | 18 |
| FBP | 339.05 | 97 | 10.423 | 18 |
| PEP | 167.05 | 79.05 | 10.477 | 15 |
| RuBP | 309.05 | 97 | 10.482 | 18 |
| HMBPP | 261.05 | 79.05 | 10.518 | 23 |
| Isocitrate | 191.05 | 73 | 10.568 | 22 |
| FMN | 455.1 | 97 | 10.593 | 27 |
| 2-Isopropylmalate | 175.05 | 115.05 | 10.603 | 18 |
| GTP | 522.1 | 159 | 10.706 | 33 |
| CTP | 482.1 | 159 | 10.729 | 36 |
| UTP | 483.1 | 159 | 10.759 | 36 |
| ATP | 506.1 | 159 | 10.801 | 40 |
| 1,3-BPG | 265.05 | 167.05 | 10.845 | 18 |
| FAD | 784.1 | 346.1 | 10.863 | 37 |
| PRPP | 389.1 | 177.05 | 10.948 | 21 |
| NADPH | 744.1 | 159.05 | 10.976 | 49 |
| PQQ | 329.1 | 241.05 | 11.051 | 15 |
| D-Camphorsulfonic acid | 231.1 | 80 | 11.055 | 32 |
| CoA | 766.1 | 408.1 | 11.105 | 30 |
| 3HB CoA | 852.1 | 772.1 | 11.109 | 41 |
| IPP,DMAPP | 245.05 | 79.05 | 11.123 | 27 |
| Malonyl-CoA | 852.1 | 808.1 | 11.125 | 27 |
| Acetyl-CoA | 808.1 | 408.1 | 11.147 | 37 |
| HMG-CoA | 910.1 | 408.1 | 11.148 | 48 |
| Crotonyl-CoA | 834.1 | 408.1 | 11.299 | 36 |
| Butanoyl-CoA | 836.1 | 408.1 | 11.373 | 37 |

Table S3 Annotated metabolites in widely targeted analysis (74 metabolites in samples were annotated using method for 121 metabolites MRM transitions, described in Table S2).

| Central metabolism | Nucleotides, nucleosides and nucleobases | Amino Acids | Others | Cofactors |
| --- | --- | --- | --- | --- |
| R5P | Cytidine | Arginine | Butanoyl-CoA | FMN |
| S7P | Adenine | Histidine | Shikimate-3P | NAD |
| F6P | Uridine | Serine | Nicotinate | NADP |
| FBP | Guanosine | Asparagine | Glutathione | FAD |
| R5P | Thymidine | Glutamate | DHAP | PQQ |
| Ru5P | ADP | Threonine | Lactate |  |
| Pyruvate | CDP | Cysteine | Pyroglutamate |  |
| Acetyl-CoA | UDP | Proline | Disaccharide-P |  |
| 3PGA | GDP | Threonine | Glycolate |  |
| 6PGA | UTP | Valine | Hexose |  |
| 1,3-BPG | ATP | Tyrosine | Sucrose |  |
| GAP | CTP | Aspartate |  |  |
| α−Glycerophosphate | GTP | Valine |  |  |
| β− Glycerophosphate | AMP | Phenylalanine |  |  |
| Orotate | TMP | Methionine |  |  |
| 2OG | XMP |  |  |  |
| Acetyl-P | UMP |  |  |  |
| Succinate | CMP |  |  |  |
| Malate | IMP |  |  |  |
| PEP | GMP |  |  |  |
| RuBP | UDP-Glu |  |  |  |
| (iso-) citrate |  |  |  |  |

Table S4 Multiple reaction monitoring (MRM) transitions for absolute quantification of CoA-related metabolites by using RP-IP-LC/QqQ-MS system [18].

| Metabolite | Precursor ion m/z of U-^12^C metabolite | Precursor ion m/z of U-^13^C metabolite | Product ion m/z of U-^12^C metabolite | Product ion m/z of U-^13^C metabolite | Target Collision Energy (V) |
| --- | --- | --- | --- | --- | --- |
| Acetyl-CoA | 808,1 | 831.1 | 408.1 | 418.1 | 15 |
| Butanoyl-CoA | 836.1 | 861.1 | 408.1 | 418.1 | 13 |
| CoA | 766.1 | 79 | 787.1 | 79 | 21 |
