# Supplementary material for: Iterative cycle of widely targeted metabolic profiling for the improvement of 1-butanol titer and productivity in Synechococcus elongatus
Source: Biotechnol Biofuels. 2018 Jul 9;11:188. doi: 10.1186/s13068-018-1187-8 (PMC6036673; doi:10.1186/s13068-018-1187-8)
Supplement: Supplementary file 2 — Additional file 2: Figure S1. Relative intensity of intracellular butanoyl-CoA, acetyl-CoA, and (iso-) citrate in BUOHSE and BUOHSE without pduP. Asterisks indicate significant differences in the strains (*: p ≤ 0.05; **: p ≤ 0.01). Figure S2. Colony PCR results for the segregation test in BUOHSE, DC7, and DC11 strain, W. T. (wild type strain), NSI (neutral site I), NSII (neutral site II). Figure S3. 1-Butanol production and cell density of S. elongatus obtained with the different RBS sequences. [file 13068_2018_1187_MOESM2_ESM.docx]

**Additional file 2**

Figure S1


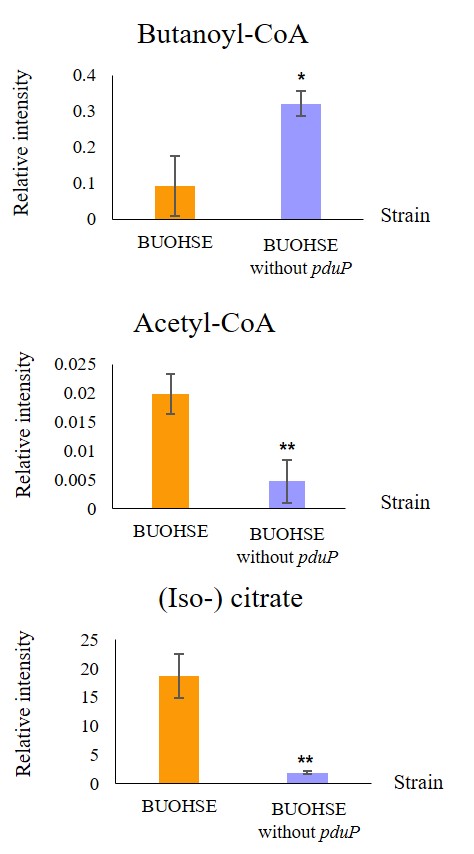


**Fig. S1** Relative intensity of intracellular butanoyl-CoA, acetyl-CoA, and (iso-) citrate in BUOHSE and BUOHSE without *pduP*. Asterisks indicate significant differences in the strains (*: p ≤ 0.05; **: p ≤ 0.01).

Figure S2


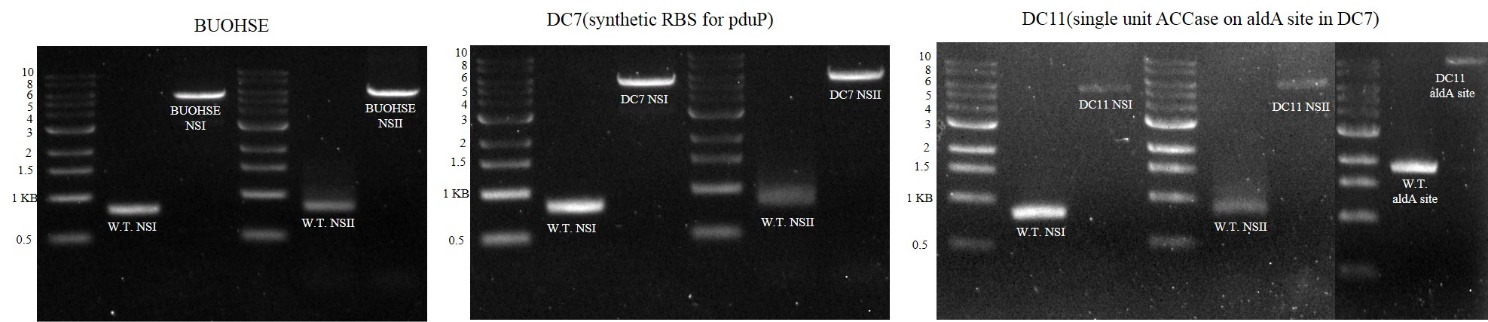


**Fig. S2** Colony PCR results for the segregation test in BUOHSE, DC7, and DC11 strain, W. T. (wild type strain), NSI (neutral site I), NSII (neutral site II).

Figure S3


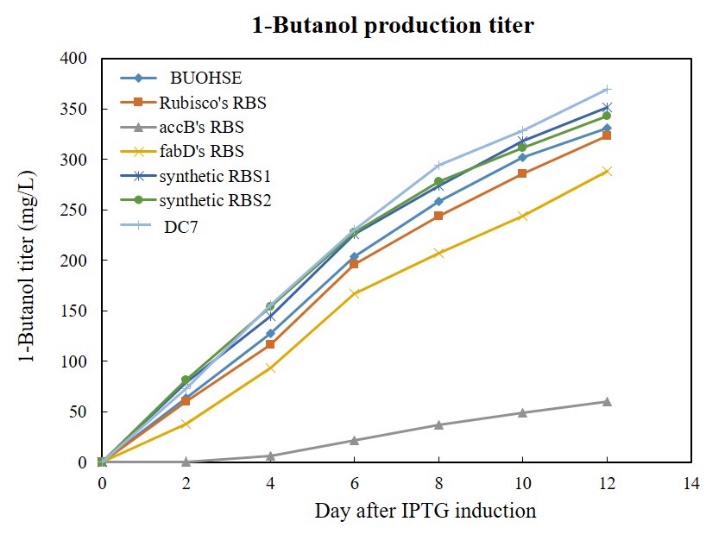

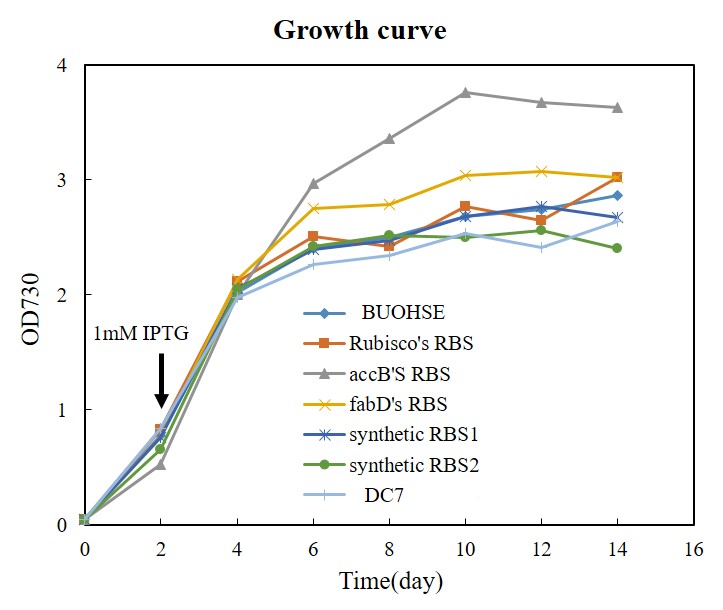


**Fig. S3** 1-Butanol production and cell density of *S. elongatus* obtained with the different RBS sequences.
